# Epidermal Interferon‐κ Drives Cutaneous Lupus‐Like Lesions, Photosensitivity, and Systemic Autoimmunity In Vivo

**DOI:** 10.1002/art.43350

**Published:** 2025-11-27

**Authors:** Benjamin Klein, Deborah J. Colesa, Yiqing Gao, Patrick A. O'Brien, Lin Zhang, Lori Lowe, Kelsey E. McNeely, Nguyen Thi Kim Nguyen, Svenja Henning, Mehrnaz Gharaee‐Kermani, Jeffrey B. Hodgin, Jacob W. S. Martens, Johann E. Gudjonsson, Celine C. Berthier, J. Michelle Kahlenberg

**Affiliations:** ^1^ University of Michigan Ann Arbor

## Abstract

**Objective:**

Keratinocyte‐derived interferon (IFN) κ is chronically overexpressed in human nonlesional systemic lupus erythematosus (SLE) skin. Recent evidence suggests that epidermal signals instruct the immune system in SLE, but whether epidermal IFNκ alone is sufficient to drive lupus phenotypes has not been investigated. This study aimed to identify whether epidermal‐specific overexpression of IFN‐κ (*Ifnk*) results in lupus‐like cutaneous and systemic inflammation.

**Methods:**

We compared 3‐month‐old (young) and 12‐month‐old (aged) Balb/c mice who overexpress *Ifnk* in the epidermis under the keratin 14 promoter (transgenic [TG]) with age‐matched Balb/c wild‐type mice and assessed local and systemic immune responses at baseline and after UV treatment. Skin lesions were assessed by histopathology, bulk RNA sequencing, and immunohistochemistry and subsequently compared to human cutaneous lupus erythematosus (CLE). Flow cytometry on lymph nodes and splenocytes at baseline and after UV treatment was performed to phenotype immune cell compositions.

**Results:**

*Ifnk* TG mice spontaneously developed CLE‐like lesions and systemic immune dysregulation. Lesions showed facial predominance, lymphocytic infiltration, immune complex deposition, and a transcriptional signature reflective of human CLE. *Ifnk* TG mice exhibited increased immune cell activation and spontaneous signs of systemic autoimmunity with higher anti–double‐stranded DNA antibodies, lymphadenopathy, and splenomegaly but lacked signs of renal inflammation. UV treatment enhanced cutaneous inflammation and splenic T cell activation in *Ifnk* TG mice.

**Conclusion:**

Together, we describe a new CLE mouse model that recapitulates features of human CLE and substantiates the role of epidermal IFNκ as a driver of CLE, photosensitivity, and systemic inflammation.

## INTRODUCTION

Epidermal keratinocytes (KCs) are critical to maintain skin homeostasis and cutaneous immunity through barrier formation and cytokine secretion.^1^ Dysfunctions in cytokine secretion pathways lead to susceptibility to infection or chronic skin inflammation, eventually accompanied by systemic inflammation. Cutaneous lupus erythematosus (CLE) is a chronic disabling autoimmune disease that can occur isolated or in association with systemic lupus erythematosus (SLE).^2^ Recent work suggests that the skin itself contributes to systemic inflammation in lupus because nonlesional skin of patients with SLE harbors a chronic type I interferon (IFN) signature, promoting recruitment of myeloid cells into the skin.^3^, ^4^, ^5^, ^6^ CLE is also characterized by strong inflammatory responses to UV light, termed photosensitivity.^7^ Our previous work identified KC‐derived IFNκ as a pivotal cytokine in lupus skin, which contributes to cutaneous inflammation and photosensitive responses by increasing apoptosis and inflammatory education of immune cells in vitro.^8^, ^9^ How an IFNκ‐rich epidermis contributes to local and systemic immune responses and whether this environment alone is sufficient to drive skin lesions and systemic disease in vivo has yet to be explored.

Here, we show that epidermal overexpression of *Ifnk* in mice leads to development of CLE‐like lesions that show significant overlap with human CLE. Furthermore, these mice develop signs of systemic autoimmunity over time. UV irradiation led to prolonged inflammatory responses in skin‐draining lymph nodes and activation of systemic T cells in *Ifnk* transgenic (TG) mice, highlighting how perturbations in the cutaneous compartment might spread systemically. Hence, we propose a new CLE mouse model by overexpression of epidermal *Ifnk* that harbors strong molecular overlap with human CLE and identifies IFNκ as sufficient to drive cutaneous lesions and systemic inflammation.

## MATERIALS AND METHODS

### Mice

*Ifnk* TG and BALB/c wild‐type (WT) mice (3 and 12 months old) were assessed for skin lesions, peripheral blood, autoantibodies, proteinuria, and immune cell composition in skin, skin‐draining inguinal lymph nodes (SDLNs), spleen, and kidney. Mice TG for *Ifnk* under the keratin 14 promoter (to promote epidermal overexpression) on the C57Bl/6 background were originally generated by Cyagen Biosciences using the PiggyBac vector and were backcrossed for more than eight generations onto the BALB/c background at the University of Michigan. Overexpression of *Ifnk* in murine skin was confirmed by genotyping and quantitative polymerase chain reaction (Supplementary Figure S1A and B). BALB/c WT mice (The Jackson Laboratory) were also bred at the University of Michigan. Mice were housed in the Unit for Animal Laboratory Medicine facility under enriched conditions at a constant temperature (22–23°C) with a 12:12‐hour light‐to‐dark cycle and optimal humidity and free access to tap water and food ad libitum. All animal procedures were performed using protocols approved by the University of Michigan Institutional Animal Care and Use Committee on Use and Care of Animals. Skin wide genome expression datasets are already available for discoid lupus erythematosus (DLE) through GEO GSE81071. Genome expression of murine skin and skin‐draining lymph nodes are now available through GEO GSE290137.

### Histology

Skin samples were fixed in 10% formalin, dehydrated, embedded in paraffin, sectioned, and stained with hematoxylin (Surgipath, 3801540, Leica Biosystems) and eosin (Surgipath, 3801600, Leica Biosystems). Hematoxylin and eosin staining was performed per standard protocols. Scoring was conducted in a blinded manner by a dermatopathologist (LL).

### RNA sequencing, analysis, and XCell enrichment tool

After RNA isolation, libraries were generated with poly‐A kits and sequencing was performed using an Illumina‐NovaSeq‐X system through the University of Michigan Advanced Genomics Core. An average 51 million reads per sample were obtained. After quality control, trimming, and read alignment, downstream analyses and normalization were performed with the DESeq2 package in R. Reads were mapped to the reference genome GRCm38 (ENSEMBL), using STAR version 2.7.8a and assigned count estimates to genes with RSEM version 1.3.3. Alignment options followed Encyclopedia of DNA Elements standards for RNA sequencing. Canonical pathways and upstream regulators from differentially expressed genes (DEGs) from the mouse model and human (GEO GSE81071) samples were identified using Ingenuity Pathway Analysis software from Qiagen. Mouse and human cell type enrichment analysis from Log2 normalized gene expression data of all samples was performed using the XCell webtool (https://comphealth.ucsf.edu/app/xcell). Heatmaps were generated using the Morpheus software from the Broad Institute (https://software.broadinstitute.org/morpheus/), and upstream regulator dot plots were generated using GraphPad Prism version 10 or an in‐house R script.

### UV irradiation

Mice were shaved and placed in a restrainer with facial protection and irradiated with 250 mJ/cm^2^ UVB three times a week for two weeks using an UV‐2 UV irradiation system (Tyler Research), which uses cascade‐phosphor UV generators that emit 310 nm of UVB radiation. Irradiated skin was outlined with a black sharpie for the two‐week observation period.

### Flow cytometry

Before processing, kidneys were cut and incubated for one hour at 37°C in tissue digestion mix (200 U/mL DNase I [Roche 4536282001], Liberase 0.1 mg/mL [Roche 540119001], and 2.4 mM CaCl_2_ in Dulbecco's modified Eagle's medium). Organs were put through a 70‐μm cell strainer in fluorescence‐activated cell sorting (FACS) buffer (3% fetal bovine serum and 1 mM EDTA) and spun at 2,000 revolutions per minute for five minutes. Lymph nodes were resuspended in FACS buffer until staining. Spleen and kidney suspensions were subjected to red blood cell lysis. Cell counts were obtained using Countess 3FL Automated Cell Counter (ThermoFisher). Single‐cell suspensions were incubated with Fc block (1:50, 1 μL per 1 million cells) for 10 minutes. Cells were stained using single‐color controls and fluorescence minus one control to assess T cells, myeloid cells, and B cells. For live/dead, heat‐killed cells (65°C for three minutes) were used as positive controls. Primary conjugated antibodies and viability dye were incubated for 30 minutes; samples were washed with phosphate buffered saline (PBS), fixed with Fluorofix Buffer solution (BioLegend, 422101), and resuspended in PBS. Samples were stored at 4°C and measured using a Cytek Aurora Spectral Analyzer. All antibodies can be found in Supplementary Table 1. Single‐color controls were used for unmixing. For spleen and lymph node cells, unstained splenocytes were used as unstained controls. For kidneys, unstained cells from kidneys were used as unstained control, and splenocytes were used for single‐color controls. Analysis of flow cytometry was performed using FlowJo version 10.10.0 and compared using GraphPad Prism version 10 (See Supplementary Materials for additional methods.)

## RESULTS

### spontaneous development of skin lesions in *Ifnk* transgenic mice

**Figure 1:**
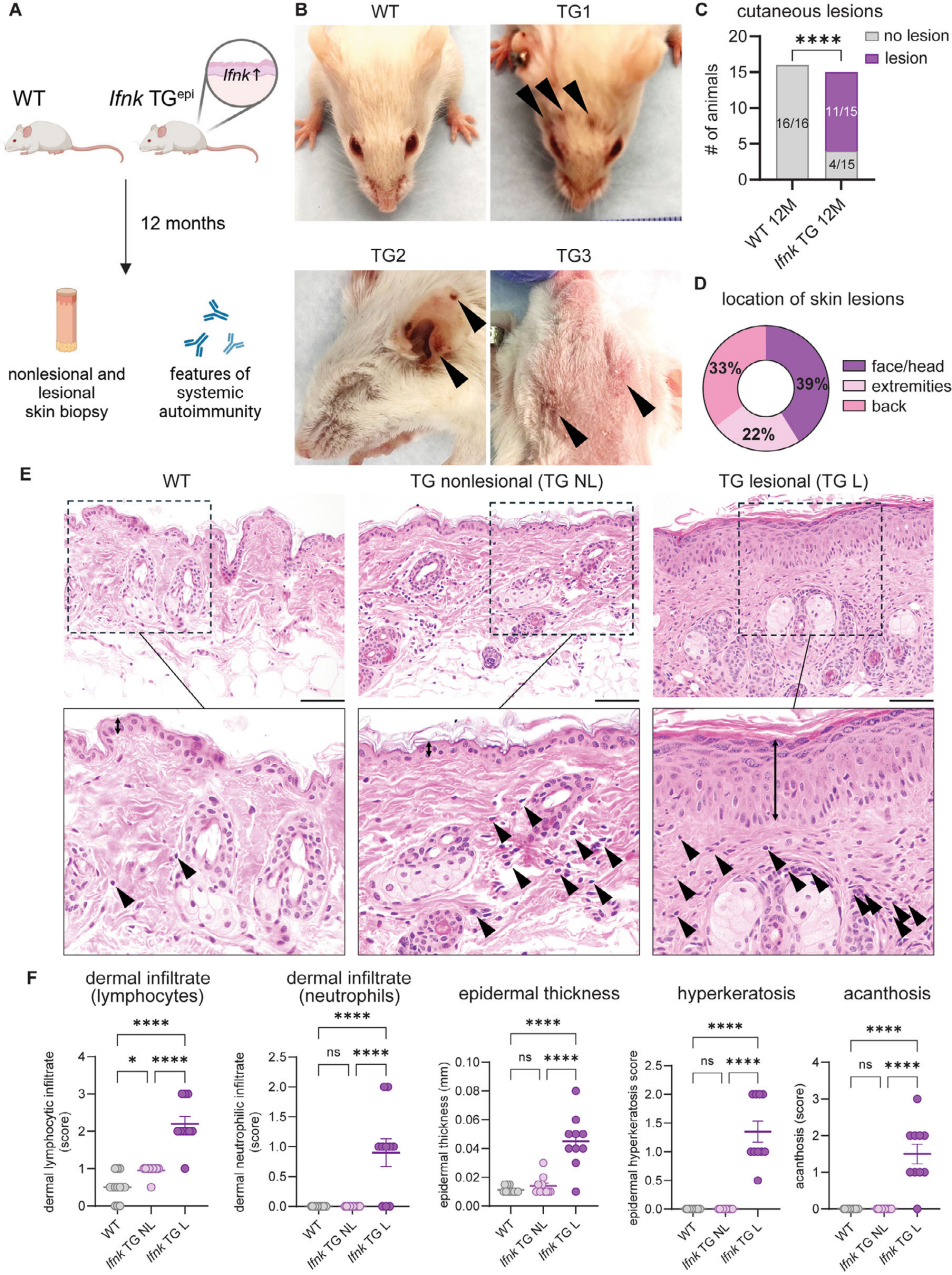
*Ifnk* TG mice develop cutaneous lupus erythematosus–like cutaneous lesions. (A) Schematic image of investigation in aged *Ifnk* TG and WT mice. (B) Representative images of WT and cutaneous lesions observed in *Ifnk* TG mice. Arrows indicate scaly plaques. (C) Quantification of cutaneous lesions at 12 months in WT and *Ifnk* TG mice, comparison by Fisher's exact test. ***P < 0.001. (D) Percentage of locations of skin lesions observed in 11 *Ifnk* TG mice. (E) Representative images of hematoxylin and eosin–stained untreated skin in WT, nonlesional *Ifnk* TG, and lesional *Ifnk* TG skin. Double‐ended arrows (↕) indicate the epidermis, and arrowheads indicate lymphocytic infiltration. Scale, 200 μm. (F) Scoring of inflammation for each indicated metric was conducted in a blinded manner by a dermatopathologist. Analysis of variance was followed by Kruskal–Wallis test. *P < 0.05; ****P < 0.0001. ns, not significant; TG, transgenic; WT, wild‐type.

Confirmation of expression of the *Ifnk* transgene (Supplementary Figure S1A and B) showed an increase of approximately two‐fold of *Ifnk* in TG mice compared to WT, which is in line with expression levels in human CLE.^8^ Mice were aged up to 12 months and assessed for survival, development of cutaneous lesions, and signs of systemic autoimmunity (Figure 1A). Survival and body weight after 12 months were significantly reduced in *Ifnk* TG mice, but overall survival remained at 81% after 12 months (Supplementary Figure S1C and D). At three months of age, *Ifnk* TG mice did not differ from WT mice in body weight, and the skin was unaffected. After 12 months, about 75% of the *Ifnk* TG mice developed cutaneous plaques located on the face, back, and occasionally on the extremities that were marked by erythema, hair loss, and the presence of slight scales, whereas no WT mice developed these lesions (Figure 1B–D; Supplementary Figure S1E). We also observed periorbital edema and swelling (Supplementary Figure S1E). Histologic examination and blinded scoring by a dermatopathologist revealed a mixed inflammatory infiltrate mostly composed of lymphocytes as well as a significant thickening of the epidermis along with acanthosis and hyperkeratosis (Figure 1E and F). Importantly, *Ifnk* TG mice exhibited increased lymphocytic infiltration in nonlesional skin compared to WT mice, which was further pronounced in lesional skin (Figure 1E and F). Of note, we observed necrotic KCs and follicular plugging, both features of human CLE (Supplementary Figure S1F).^2^ Some lesions exhibited neutrophilic infiltrates, which can be observed in a significant subset of CLE manifestations.^10^ MX1, an IFN‐induced gene, was significantly increased in nonlesional epidermis of *Ifnk* TG mice compared to WT, consistent with a chronic type I IFN signature (Supplementary Figure S1G and H). These results indicate that overexpression of epidermal *Ifnk* alone is sufficient to drive development of cutaneous inflammation resulting in lesion development.

### Characterization of transcriptomic signatures of lesional skin in *Ifnk* TG mice

**Figure 2:**
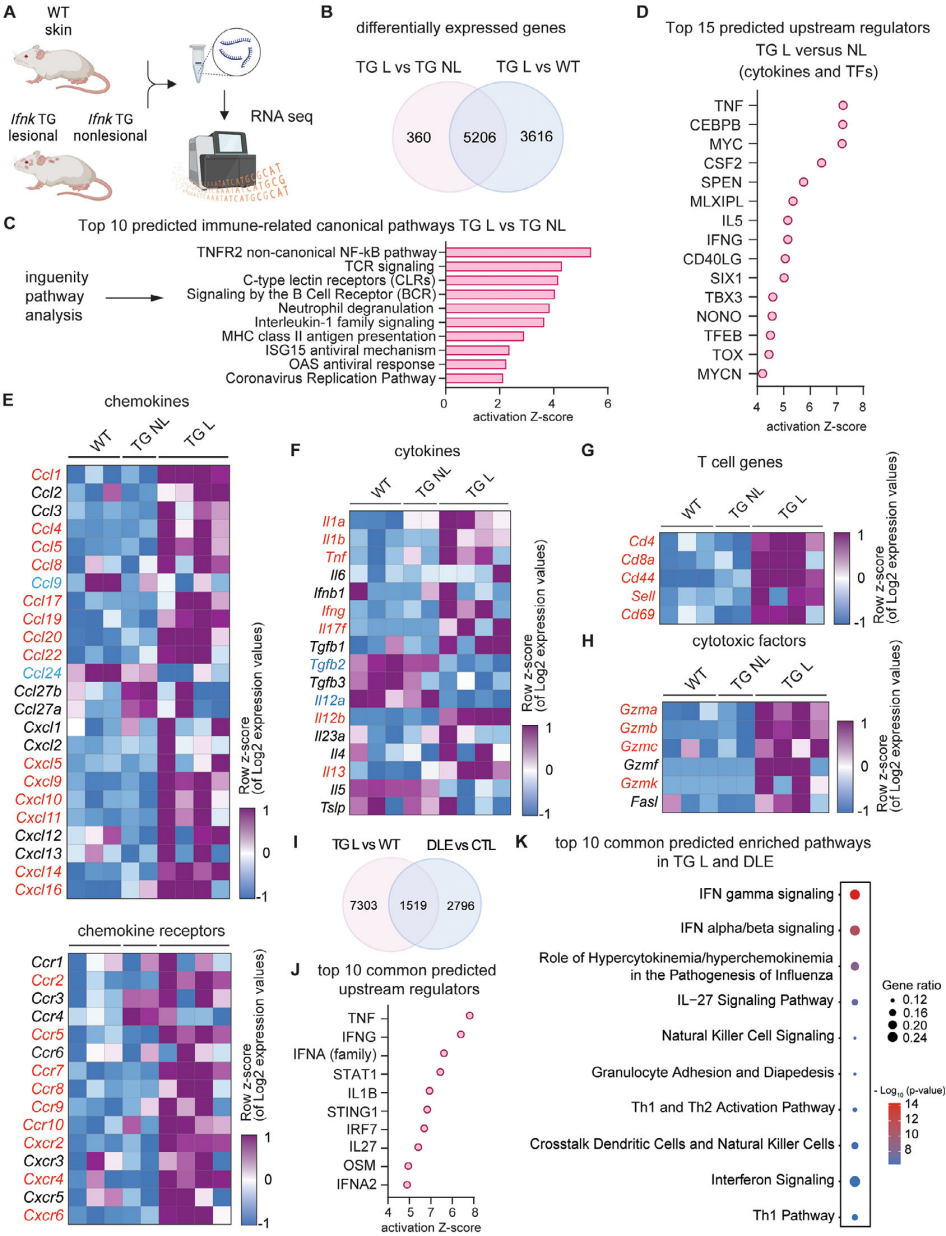
Transcriptional overlap of skin lesions in *Ifnk* TG mice with human cutaneous lupus erythematosus. (A) Experimental approach using 12‐month‐old WT and *Ifnk* TG mice. (B) Differentially expressed genes (DEGs) between *Ifnk* TG nonlesional (TG NL) and TG lesional (TG L) skin compared to wild‐type (WT). (C) Bar plot representation of the top 10 predicted activated immune‐related canonical pathways enriched among DEGs in *Ifnk* TG L skin versus *Ifnk* TG NL skin. (D) Dot plot of the top 15 predicted upstream regulators (cytokines and transcription factors) enriched in DEGs in *Ifnk* TG L skin versus *Ifnk* TG NL skin. (E–H) Heatmaps of selected genes for chemokines and (E) chemokine receptors, (F) cytokines, (G) T cell–related genes, and (H) cytotoxic factors. Red‐colored font indicates significant up‐regulation in *Ifnk* TG L versus WT, whereas blue‐colored font indicates significant down‐regulation in *Ifnk* TG L versus WT. (I) Number of shared and distinct DEGs in *Ifnk* TG L skin versus WT with human DLE versus CTL skin. (J) Dot plot showing activation Z‐scores of the top 10 predicted upstream regulators enriched in common DEGs. (K) Bar plot showing top 10 predicted activated pathways enriched among shared DEGs between *Ifnk* TG L versus WT and DLE versus CTL skin. CTL, control; DLE, discoid lupus erythematosus; IFN, interferon; IL, interleukin; ISG, IFN‐stimulated gene; MHC, major histocompatibility complex; OAS, 2′,5′‐oligoadenylate synthetase; RNA seq, RNA sequencing; TCR, T cell receptor; TF, transcription factor; TG, transgenic; TNFR, tumor necrosis factor receptor; WT, wild‐type.

Because these lesions had histologic features of CLE, we next wanted to transcriptionally compare them to human CLE lesions. Bulk RNA sequencing of WT and lesional and nonlesional *Ifnk* TG skin was completed as shown in Figure 2A. We identified 8,822 DEGs in *Ifnk* TG skin compared to WT skin (Figure 2B). *Ifnk* TG skin showed high expression of IFN‐related genes compared to WT. We then performed pathway analysis of lesional versus nonlesional skin in *Ifnk* TG mice to evaluate lesion progression. This comparison revealed cell cycle– and keratinization‐related pathways to be the most nonimmune up‐regulated pathways, reflective of hyperkeratosis and acanthosis. Top immune‐related pathways in *Ifnk* TG lesional versus nonlesional were related to NF‐κB, T cell receptor/B cell receptor signaling, antigen presentation, neutrophil degranulation, and antiviral responses (Figure 2C). Top upstream regulators of lesional skin included several cytokines known to be involved in CLE pathogenesis, such as tumor necrosis factor (TNF) and IFNγ (Figure 2D; Supplementary Figure S2A). Next, we looked at chemokines and cytokines and their respective receptors that contribute to the inflammatory infiltrate observed both in nonlesional and lesional *Ifnk* TG skin (Figure 2E and F; Supplementary Figure S2B). In nonlesional skin, we observed significant up‐regulation of the ligand–receptor pair *Ccl27/Ccr4*, which contribute to T cell homing in the skin (Figure 2E).^11^ In addition, *Cxcl16* was increased, which is known to promote myeloid cell recruitment.^12^, ^13^ Lesional skin showed strong up‐regulation of *Cxcl9*, *Cxcl10*, and *Cxcl11*, three major chemokines implicated in CLE pathogenesis.^14^, ^15^ Up‐regulation of *Ccl20* and *Ccl5* was also present, both of which are increased in human CLE lesions (Figure 2E).^16^ Analysis of chemokine receptors revealed significant up‐regulation of C‐C chemokine recepotors (*Ccr2*, *Ccr5*, *Ccr7*) and C‐X‐C motif chemokine receptors (*Cxcr2*, *Cxcr4*, *Cxcr6*), all of which have been observed to be up‐regulated in human CLE (Figure 2E).^17^ We found prominent cytokine differences, including expression of *Ifng*, *Il1a*, *Il1b*, and *TNF* in cutaneous lesions, reflective of a dominant Th1‐biased immune response, as seen in human CLE (Figure 2F).^14^ Furthermore, cutaneous lesions exhibited a prominent IFN signature, with multiple IFN‐stimulated genes being significantly up‐regulated (Supplementary Figure S2B). These results suggest overexpression of *Ifnk* drives spontaneous development of CLE‐like lesions and that a strong activation of type I and II IFN signaling exists in these lesions, similar to human CLE. T cell markers *Cd4* and *Cd8a* and the T cell activation marker *Cd69* were significantly up‐regulated in lesional skin (Figure 2G). Granzymes, including *Gzma* and *Gzmb*, were enriched in lesional skin, reflective of a cytotoxic T cell response (Figure 2H). Because murine lesions were mostly located on the face and marked by hair loss and chronicity, we next explored the transcriptional overlap with human DLE lesions using our previously published dataset of 47 patients.^18^ Here, we saw a total of 1,519 genes overlapping in DLE and lesional *Ifnk* TG skin (Figure 2I), which were characterized by common upstream regulators such as TNF, IFNγ, IFNα, STAT1, interleukin (IL)‐1β, and stimulator of IFN genes, reflecting strong molecular similarity between lesional *Ifnk* TG skin and DLE (Figure 2J). Pathway analysis revealed enrichment in IFN signaling, T cell activation, and immune cell crosstalk (Figure 2K), all hallmarks of CLE. Together, the transcriptional signature of lesional *Ifnk* TG skin is CLE‐like, shares substantial overlap with human CLE, and suggests IFNκ promotes T cell activation and inflammatory cytokine production.

### CLE‐like lesions characterized by myeloid and T cell infiltration

**Figure 3:**
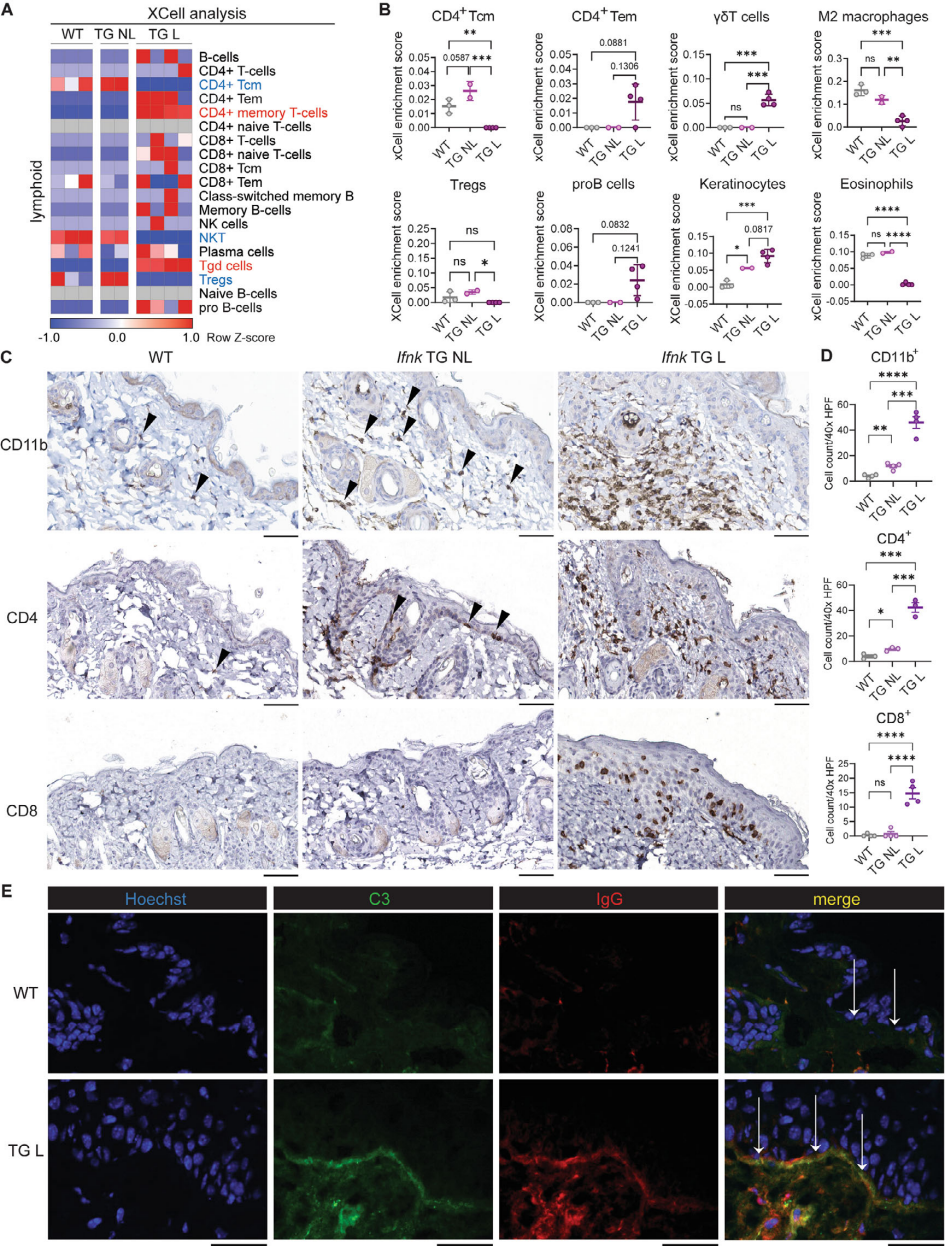
Cutaneous lupus erythematosus–like lesions exhibit myeloid and T cell infiltration. (A) Heatmap of XCell analysis representing XCell‐derived row Z‐scores for lymphoid cell type in WT, *Ifnk* TG NL, and *Ifnk* TG L skin. Red‐colored font indicates significant up‐regulation in *Ifnk* TG L skin versus WT, whereas blue‐colored font indicates significant down‐regulation in *Ifnk* TG L skin versus WT. (B) Scatter plots of XCell scores for CD4^+^ γδ central memory T (Tcm), effector memory T (Tem), cells, M2 macrophages, Treg cells, pro B cells, keratinocytes, and eosinophils. *P < 0.05; **P < 0.01; ***P < 0.001; ****P < 0.0001. (C) Representative images of immunohistochemistry of WT, *Ifnk* TG NL, and *Ifnk* TG L skin stained for CD11b, CD4, and CD8. Arrows indicate positive staining in nonlesional skin. Scale, 60 μm. (D) Quantification of cell counts of staining shown in (C) per 40× high‐power field (n = 3 to 4 per staining). *P < 0.05; **P < 0.01; ***P < 0.001; ****P < 0.0001. (E) Representative images of immune complex deposition at the dermoepidermal junction zone (arrows) in WT (n = 3) and TG L skin (n = 4) by staining of frozen sections for C3 (green), IgG (red), and Hoechst (blue). Scale, 50 μm. Student's *t*‐test or Mann–Whitney U test. ns, not significant; TG, transgenic; TG L, TG lesional; TG NL, TG nonlesional; WT, wild‐type.

To characterize the cellular infiltrate in lesional *Ifnk* TG skin, we performed XCell analysis, an in silico approach that uses validated gene signatures to deduce the underlying cell types in our transcriptional dataset.^19^ We observed significant enrichment in T cells with a shift toward CD4^+^ T memory cells with low scores for central memory T cells but high scores for effector memory T (Tem) cells, suggesting mature T cell–driven inflammatory responses in lesional skin (Figure 3A and B). In the myeloid compartment, we observed enrichment for neutrophils, monocytes, and dendritic cells (DCs) but were underpowered to see statistical significance (Supplementary Figure S2C). Lesions also exhibited higher scores for KCs and γδ T cells and reduced scores for Treg cells, M2 macrophages, and eosinophils (Figure 3A and B). Interestingly, scores for B cells, which have recently been appreciated to be enriched in human DLE subtype of CLE,^20^ were enriched in *Ifnk* TG lesional skin (Figure 3A and B). To confirm the cellular signatures in *Ifnk* TG lesional skin, we performed immunohistochemistry of myeloid marker CD11b, which revealed strong dermal infiltration in lesional skin (Figure 3C and D). Nonlesional skin of *Ifnk* TG mice also showed enrichment of CD11b^+^ cells compared to WT skin (Figure 3C and D), indicating myeloid cell recruitment in nonlesional skin of *Ifnk* TG mice. Interestingly, CD4 T cells were also enriched in nonlesional skin close to basal KCs (Figure 3C and D), in line with our previous observation showing normal‐appearing skin exhibits up‐regulation of genes involved in T cell homing. Skin lesions exhibited significant CD8 T cell enrichment along the dermoepidermal junction (DEJ) and had substantial dermal CD4 T cell infiltrates, in line with human DLE. The CD4 to CD8 ratio was between ~1.5 and ~2.5, which has been reported in human DLE as compared to other subtypes of CLE such as subacute CLE.^21^ Moreover, lesions in *Ifnk* TG mice exhibited signs of immune complex deposition at the DEJ (Figure 3E), an important feature of human CLE.^2^ Nonlesional skin of two‐thirds of *Ifnk* TG mice also had positive staining at the DEJ (Supplementary Figure S2D), in line with the lupus band detectable in nonlesional skin in human SLE.^22^ Together, lesions in *Ifnk* TG mice show strong transcriptional and cellular overlap with human DLE, and nonlesional skin recapitulates findings that are observed in human SLE.

### Systemic inflammation resembling SLE in *Ifnk*‐expressing mice

**Figure 4:**
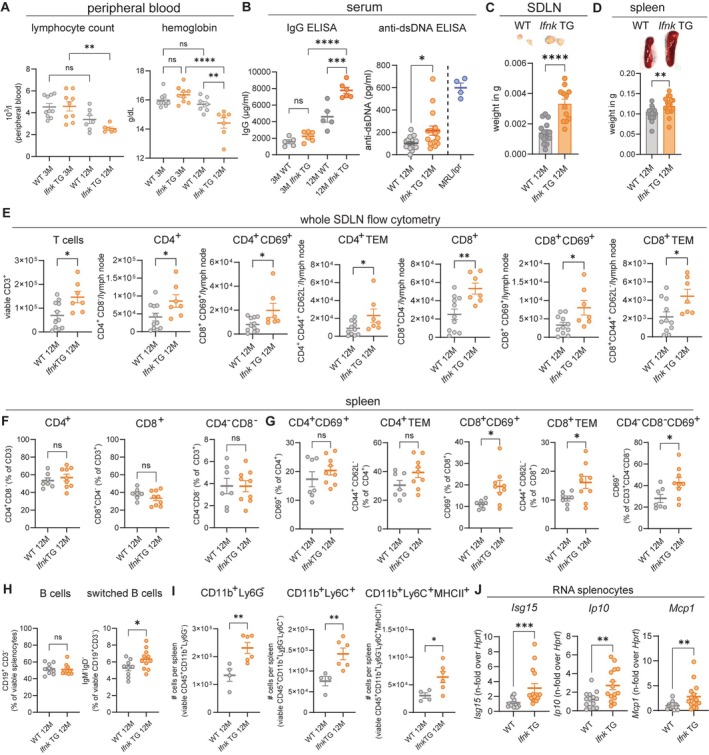
*Ifnk* TG mice exhibit signs of systemic autoimmunity. (A) Amounts of lymphocytes and hemoglobin in young (3 months [3M] of age) and old (12 months [12M] of age) WT and *Ifnk* TG mice (n = 6–9 per group). **P < 0.01; ****P < 0.0001. (B) Serum total IgG (n = 5–6 per group) and anti‐dsDNA antibodies (n = 8–16 per group) with MRL/lpr mice (n = 4) as positive control. *P < 0.05; ***P < 0.001; ****P < 0.0001. Vertical dashed line is dividing MRL/lpr serum (used as a positive control) from the experimental groups.  (C) Representative picture of skin‐draining lymph node (SDLN) and weights/node in WT and *Ifnk* TG mice. ****P < 0.0001. (D) Representative image of spleens and weights in WT and *Ifnk* TG mice. **P < 0.01. (E) Results of total cells per SDLN of WT and *Ifnk* TG mice in spectral flow cytometry. Gating strategies are shown in Supplementary Figure S3. *P < 0.05; **P < 0.01. (F and G) Percentages of T cell subpopulations in spleens of WT and *Ifnk* TG mice. *P < 0.05. (H) Percentages of B cell subsets. *P < 0.05. (I) Total splenic myeloid subsets in WT and *Ifnk* TG mice (n = 4–6 per group). **P < 0.01. (J) Relative expression of *Isg15*, *Ip10*, and *Mcp1* in WT and *Ifnk* TG mice of both sexes, normalized to *Hprt*. Sex‐specific expression of these genes is shown in Supplementary Figure S3J. **P < 0.01; ***P < 0.001. Analysis of variance was followed by (A and B) Sidak's multiple comparisons test and (C–M) Student's *t*‐test/Mann–Whitney U test (n ≥ 3 per experimental condition for WT and *Ifnk* TG mice). dsDNA, double‐stranded DNA; ELISA, enzyme‐linked immunosorbent assay; MHC, major histocompatibility complex; ns, not significant; TEM, effector memory T; TG, transgenic; WT, wild‐type.

Hallmarks of SLE include changes in peripheral blood, production of autoantibodies, and potential life‐threatening renal involvement. Therefore, we aimed to characterize systemic immune changes in the *Ifnk* TG versus WT mice over time. We first obtained complete blood count (CBC) levels through submandibular bleedings from mice at 3 and 12 months of age. Importantly, *Ifnk* TG, but not WT, mice exhibited significant decreases of peripheral lymphocyte count and hemoglobin levels (Figure 4A), in line with hematologic abnormalities observed in human SLE.^23^ Blood smears were examined for morphology of lymphocytes and red blood cells (eg, schistocytes, spherocytes), revealing no abnormalities at 3 and 12 months (Supplementary Figure S3A). Strikingly, *Ifnk* TG mice exhibited significantly higher levels of total serum IgG levels and higher anti–double‐stranded DNA (dsDNA) antibodies compared to WT (Figure 4B). Moreover, *Ifnk* TG mice exhibited enlarged SDLNs and increased spleen weights (Figure 4C and D; Supplementary Figure S3B–D), characteristics often observed in human and murine SLE. We then characterized the immunologic landscape of both lymph nodes and spleens of *Ifnk* TG mice by flow cytometry (for gating, see Supplementary Figure S3E–H). Enlarged SDLNs were higher in CD69^+^CD4^+^ and CD8^+^ T cells (Figure 4E). CD44^+^CD62L^−^ Tem cells were also enriched compared to WT (Figure 4E). Furthermore, SDLNs showed significantly higher amounts of double‐negative T cells that showed higher amounts of cells positive for CD69 (Supplementary Figure S3F). Flow cytometry of the spleen showed no increased percentage of CD4^+^, CD8^+^, or double‐negative T cells (Figure 4F), but *Ifnk* TG mice exhibited higher percentages of CD8^+^CD69^+^ and CD8^+^CD44^+^CD62L^−^ Tem cells, whereas these populations had similar percentages in CD4^+^ T cells (Figure 4G). Percentages of B cells were not overall increased in *Ifnk* TG mice compared to WT, but we observed higher rates of switched B cells, in line with increased abundance of autoantibodies (Figure 4H). Investigations of myeloid cells revealed increased total amounts of CD11b^+^Ly6G^−^, CD11b^+^Ly6G^−^Ly6C^+^, and classical DCs (major histocompatibility complex [MHC] II^+^CD11c^+^; Figure 4I; Supplementary Figure S3I). Furthermore, we saw significantly more CD11b^+^Ly6G^−^Ly6C^+^MHCII^+^ cells (Figure 4I), which supports activation and antigen presentation as a phenotype of myeloid cells in *Ifnk* TG mice. We also examined gene expression in the spleen for IFN‐regulated and inflammatory genes and found increased expression of *Isg15*, *Ip10*, and *Mcp1* in *Ifnk* TG mice (Figure 4J; Supplementary Figure S3J), indicative of an IFN‐skewed splenic phenotype. Together, these data support that aged *Ifnk* TG mice develop signs of systemic autoimmunity, indicating that chronic overexpression of epidermal type I IFN contributes to systemic immune responses.

### Renal inflammation in *Ifnk* TG mice

**Figure 5:**
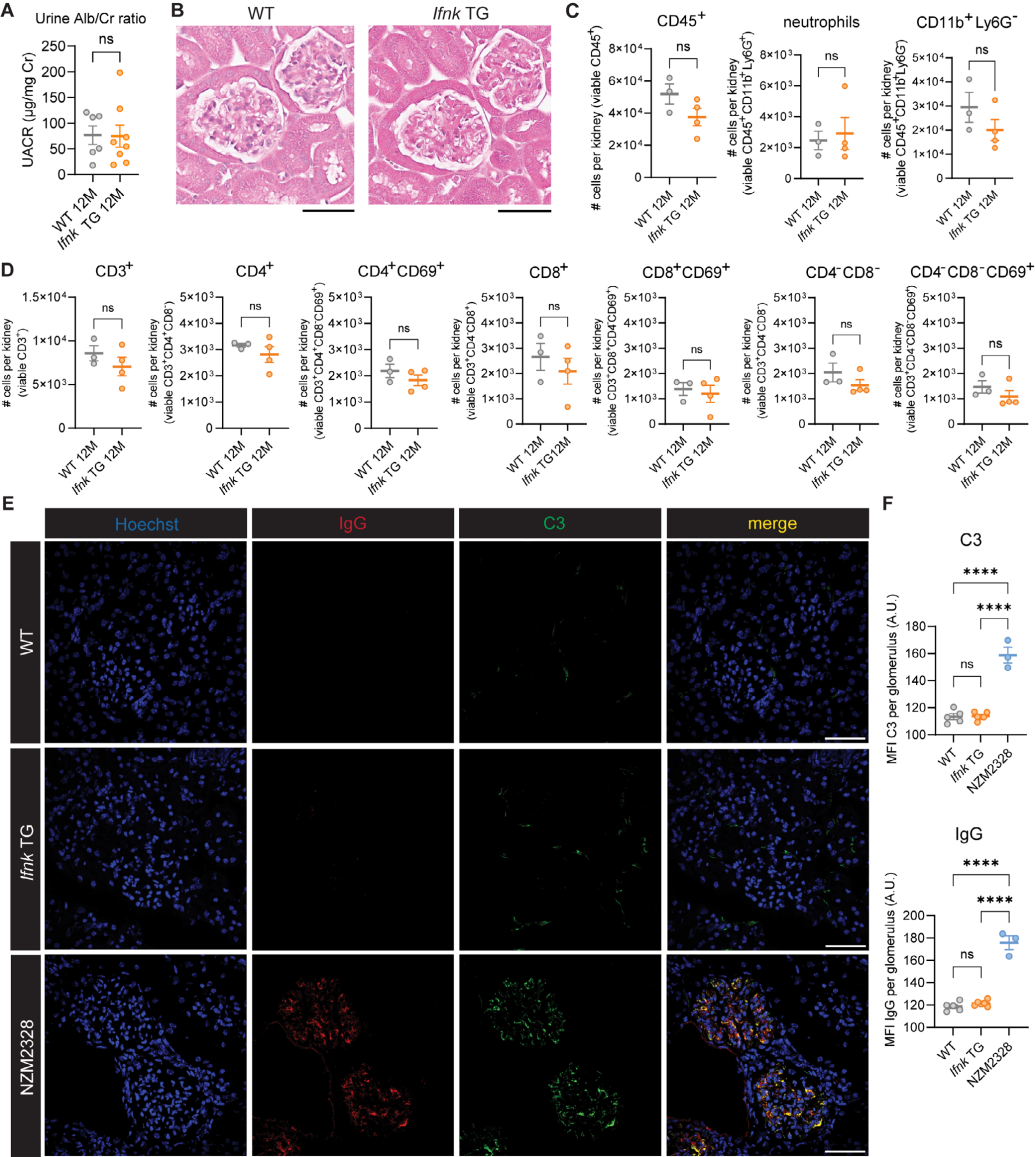
Overexpression of epidermal *Ifnk* does not drive renal inflammation. (A) Urine albumin to creatinine (Alb/Cr) ratio (in micrograms per milligram) in urine obtained from WT and *Ifnk* TG mice at the day of euthanasia (12M). (B) Representative images of hematoxylin and eosin–stained kidneys in WT and *Ifnk* TG mice. Blinded analysis of a renal pathologist revealed no differences in glomerular and interstitial pathology. Scale, 60 μm. (C and D) Flow cytometry of tissue‐resident immune cells in heparin‐perfused kidneys to assess immune cell infiltration via (C) CD45^+^ cells and myeloid cells and (D) T cells and T cell subsets. (E) Representative images of tissue immunofluorescence of frozen sections from heparin‐perfused kidneys from WT, *Ifnk* TG, and NZM2328 mice stained for nuclei (DAPI; blue), IgG (red), and C3 (green). Scale, 50 μm. (F) Mean fluorescence intensity (MFI) of tissue immunofluorescence C3 and IgG in WT (n = 5), *Ifnk* TG (n = 6), and NZM2328 (n = 3) mice. For MFI per glomerulus, average intensity values were obtained after quantification of 10 glomeruli from three different slide locations per mouse. ****P < 0.0001. 12M, 12 months; A.U., arbitrary units; ns, not significant; TG, transgenic; UACR, urine albumin to creatinine; WT, wild‐type.

Given systemic immune cell activation and dsDNA antibody positivity, we then investigated whether *Ifnk* TG mice develop renal inflammation. Notably, *Ifnk* TG mice did not develop proteinuria (Figure 5A) or glomerular pathology (Figure 5B). Flow cytometry of heparin‐flushed kidneys did not show differences in total CD45^+^ cells, CD11b^+^Ly6G^−^ cells, neutrophils, T cells, or T cell subsets (Figure 5C and D), suggestive of absent renal immune cell infiltration. Kidney sections from WT, *Ifnk* TG, and lupus‐prone NZM2328 mice were examined for glomerular immune complex deposition (Figure 5E). Although the latter exhibited robust IgG and C3 staining, there was no evidence of renal immune complex deposition in WT or *Ifnk* TG mice (Figure 5E and F). These results show that despite signs of systemic autoimmunity, chronically increased epidermal IFNκ is not sufficient to drive activation of renal disease in the BALB/c background.

### Local and systemic T cell responses after UVB treatment in *Ifnk* TG mice

**Figure 6:**
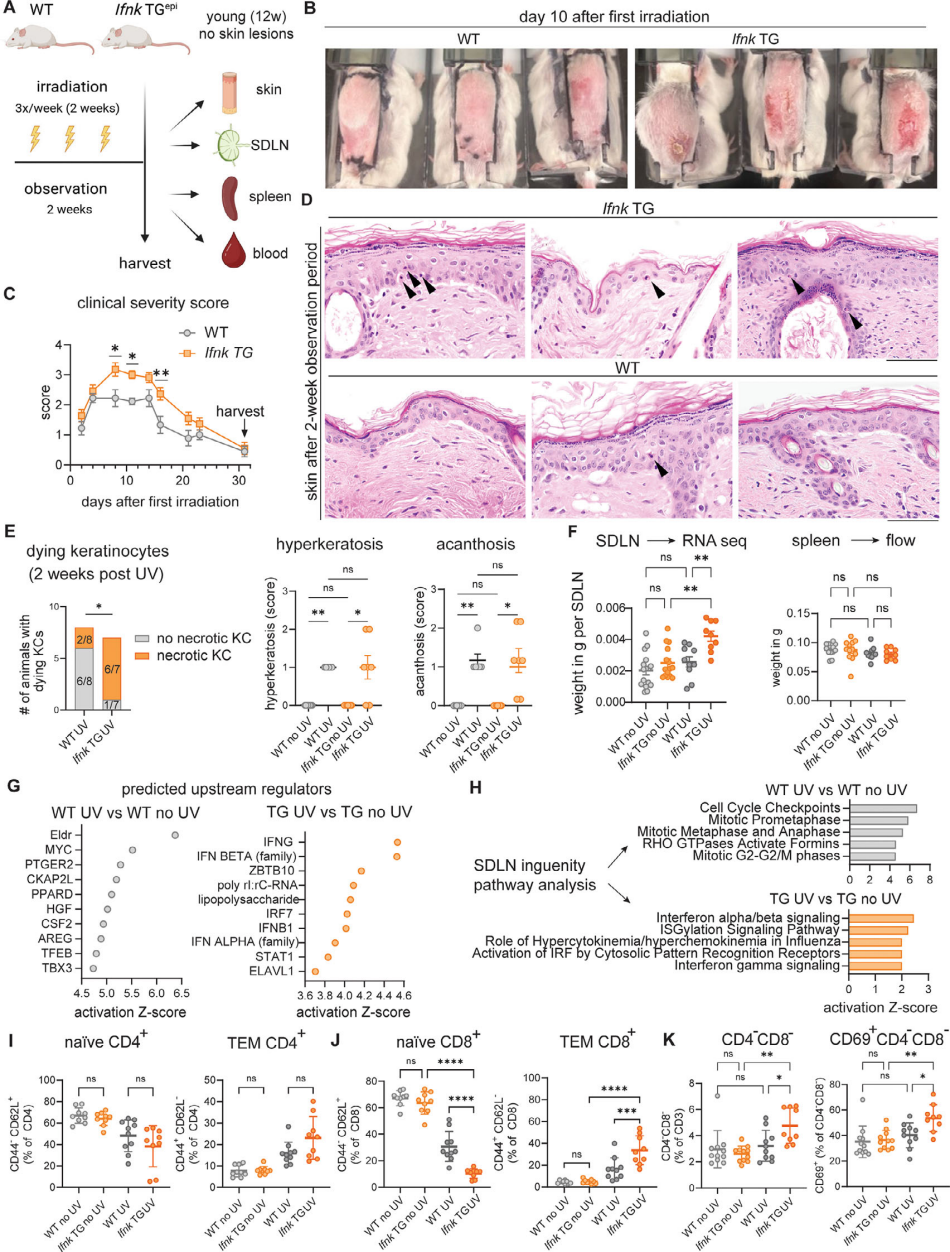
IFNκ promotes photosensitivity and systemic T cell activation in response to UVB in vivo. (A) Experimental procedure for chronic UVB‐induced skin inflammation. (B) Representative images of UV‐induced skin lesions in WT and *Ifnk* TG mice at the last day of irradiation. Note erythema and fissures in *Ifnk* TG mice. (C) Clinical severity score over time of irradiation and observation. *P < 0.05; **P < 0.01. (D) Representative images of hematoxylin and eosin—stained, UV‐irradiated skin at time of harvest (after observation period). Arrowheads indicate apoptotic/necrotic KCs. (E) Number of animals with histologically dying KCs, with Fisher's exact test performed. *P < 0.05; **P < 0.01. (F) Weight per SDLN in WT and *Ifnk* TG mice at baseline and after UVB. **P < 0.01. (G) Dot plot of SDLN bulk RNA sequencing (RNA seq) shows top upstream regulators of UV response enriched in each strain. (H) Bar plot showing the activation Z‐scores of the top five upstream regulators enriched after UV in each strain. (I and J) Splenic naive and TEM (I) CD4^+^ T cells and (J) CD8^+^ T cells before and after UV treatment. ***P < 0.001; ****P < 0.0001. (K) Splenic double‐negative T cells and CD69^+^CD4^−^CD8^−^ cells (n = 7 to 9 per experimental condition). *P < 0.05; **P < 0.01. One‐/two‐way analysis of variance was followed by Sidak's multiple comparisons test. epi, epidermal; IFN, interferon; IRF, IFN regulatory factor; KC, keratinocyte; ns, not significant; SDLN, skin‐draining inguinal lymph node; TEM, effector memory T; TG, transgenic; WT, wild‐type.

One important feature of human CLE is photosensitivity, characterized by enhanced epidermal cell death and increased inflammatory responses to UV light that can result in systemic inflammation.^2^, ^7^ We previously showed that *Ifnk* TG mice exhibit increased epidermal cell death after short‐term UV treatment.^24^ In murine lupus models, long‐term UV treatment results in a type I IFN–dependent myeloid cell recruitment into the skin, T cell activation, and depletion of regulatory T cells in SDLNs.^25^, ^26^ To see whether *Ifnk* overexpression was sufficient to drive this phenotype, we irradiated WT and *Ifnk* TG mice with long‐term UVB treatment (three times a week for two weeks) and observed for an additional two weeks to allow for immune activation before harvesting skin, SDLNs, peripheral blood, spleens, and kidneys for further analysis (Figure 6A). Neither *Ifnk* TG mice nor WT mice died during this experiment. Notably, *Ifnk* TG mice exhibited enhanced cutaneous severity scores from day 8 to 15 after UV treatment but were able to heal gross skin lesions (Figure 6B and C; Supplementary Figure S4A). Surprisingly, enhanced KC cell death was still noted histologically in *Ifnk* TG mice two weeks after UV treatment was completed, suggesting chronic activation of cell death pathways in *Ifnk* TG mice even after macroscopic lesions had healed (Figure 6D and E), similar to human CLE.^27^ SDLNs were significantly enlarged in UV‐treated *Ifnk* TG versus WT mice (Figure 6F). Bulk RNA sequencing of SDLNs revealed upstream regulators in WT mice involved in cell cycle regulation (ELDR, MYC)^28^, ^29^ and post‐UV immunosuppression (PTGER2),^30^, ^31^ whereas *Ifnk* TG mice had type I/II IFNs as the most significant upstream regulators of transcriptional changes (Figure 6G). Similarly, pathway analysis was significant for IFN signaling in *Ifnk* TG mice; pathways involved in cell cycle were enriched in WT mice, suggesting a chronic IFN‐mediated proinflammatory response in *Ifnk* TG mice versus WT (Figure 6H; Supplementary Figure S4B). Given that UV treatments in lupus‐prone mice can trigger systemic immune activation, we then examined systemic response to UV treatment. Evaluation of peripheral blood before and after UV treatment showed a significant reduction of hemoglobin in *Ifnk* TG mice but not in WT (Supplementary Figure S4C). Immunophenotyping of splenocytes assessed by flow cytometry revealed a significant immune activation similar to what was seen in aged *Ifnk* TG mice. This included shifts of naive (CD62L^+^CD44^−^) CD8^+^ T cells toward Tem (CD44^+^CD62L^−^) CD8^+^ cells in *Ifnk* TG versus WT mice after UV treatment (Figure 6I–K). Splenic CD4^+^ T cells showed a similar trend without significance (Figure 6J). Furthermore, *Ifnk* TG double‐negative T cells were enriched in CD69 (Figure 6K). Both female and male *Ifnk* TG mice exhibited this skewing toward effector CD8^+^ T cell responses, indicating this is independent of murine sex differences. Together, these results indicate cutaneous and systemic photosensitive responses in *Ifnk* TG mice and suggest that overproduction of epidermal IFNκ promotes systemic CD8^+^ Tem cell maturation after UVB treatment.

## DISCUSSION

Epidermal type I IFN primes the skin for photosensitivity, enhanced UVB‐induced epidermal cell death, resulted in metabolic reprogramming of myeloid cells during innate immune stimulation, and activated DCs.^8^, ^24^, ^32^, ^33^ In this report, we describe a new IFNκ‐induced murine CLE model with features of systemic autoimmunity but absence of renal inflammation. Mice with overexpression of epidermal *Ifnk* develop skin lesions that share morphologic, transcriptional, and cellular overlap with human DLE and exhibited signs of SLE‐like disease. Together, our results provide mechanistic support for recent translational evidence that a type I IFN–rich epidermis results in cutaneous inflammation that can further promote systemic autoimmunity.

Studying CLE in mice has been a challenge. Most evidence of murine CLE is derived from studying mice with SLE features that also develop skin lesions. The MRL/*lpr* model is the most popular with development of skin manifestations in one‐third of animals at three to six months of age, in parallel to kidney inflammation.^34^ This is accompanied by excessive lymphoproliferation and splenomegaly, which often causes death of the animal. The suitability of this mouse model to study CLE is questionable because CLE can occur on its own and progresses into SLE only in 5%–25% of mice. Furthermore, MRL/*lpr* mice do not exhibit robust IFN signatures or show involvement of B cells in lesions, which has been described as a distinct feature of human DLE.^20^ Other murine models include the global PD‐1H (VISTA) knockout mouse, which develops mild cutaneous inflammation at approximately six months of age, together with pericardial calcification, general immune overactivation, and formation of autoantibodies.^35^ Additionally, there are more complicated models requiring deletion of *Tlr9* with ovalbumin (OVA) expression that undergo adoptive T cell transfer with OVA‐specific DO11 T cells into sublethally irradiated mice.^36^

There is mounting evidence for the role of KC dysfunction in both onset and flare of CLE and SLE^4^, ^37^: Epidermal‐specific loss of PPARγ was shown to result in lupus‐like disease characterized by epidermal type I IFN secretion, resulting in immune cell recruitment and B cell activation with production of autoantibodies.^6^ Furthermore, another model investigated epidermal overexpression of female‐biased transcription factor VGLL3, which resulted in onset of a severe lupus‐like rash and systemic B cell expansion.^38^ These models highlight the important role of KCs in promoting systemic immune responses. The role of IFNκ signaling in these models would require additional investigations and crosses to *Ifnk* knockout mice. Our previous work demonstrated that KCs in nonlesional lupus skin chronically up‐regulate type I IFN,^3^, ^8^, ^39^ which we recapitulate with the epidermal‐specific *Ifnk* TG strain. This model has some useful features, including delayed onset of autoimmunity, which provides time to study triggers of immune system activation. Of note, IFNα treatment via adenovirus can drive acceleration of lupus nephritis in lupus‐prone mice,^40^ but IFNκ alone does not drive renal inflammation in our model. This is interesting because it suggests that immune activation (eg, production of autoantibodies) is not sufficient for nephritis. Whether this means that a second hit is required or whether direct type I IFN overexpression in the renal tissues, such as the endothelium or tubular cells, is required remains to be determined. Certainly, something is needed to coax innate immune cells into the kidney to initiate disease. Lupus nephritis onset is associated with markers of monocyte/neutrophil degranulation and macrophage activation in human SLE.^41^ Also, activation of innate lymphoid cells was shown to be critical to result in nephritis in lupus‐prone mice.^42^

Apart from KCs, myeloid cells have been shown to express IFNκ in lupus skin disease.^43^ We did not dissect the role of other cell types expressing IFNκ in our model. Nonlesional skin exhibits up‐regulation of the type I IFN signal only in the epidermis; however, lesional skin likely exhibits a chronic autocrine IFN‐producing loop with contributions from myeloid cells, which has been shown in human CLE.^39^, ^43^ Another possibility for crosstalk with the systemic immune system could be transfer of KC‐derived messenger RNA (mRNA) to Langerhans cells (LCs).^44^ Hence, transfer of *Ifnk* mRNA by LCs from surrounding KCs might promote immune dysregulation in this model. Further studies will elucidate the role of IFNκ in other cell types in lupus skin.

UV‐induced skin inflammation is enhanced in a type I IFN–rich environment.^8^, ^33^, ^39^ Other cytokines that contribute to UV‐induced skin inflammation are IL‐6 and TNFα.^45^, ^46^ The dependency of systemic and cutaneous phenotypes on these cytokines were not assessed in this study. Whether abrogation of type I IFN alone in the skin is sufficient to completely block UV‐induced skin inflammation remains a question for future research. Importantly, KC‐derived production of IFN participates both in local and systemic immune activation after UV treatment. Indeed, lupus‐prone mice exhibit early type I IFN–dependent cutaneous recruitment of myeloid cells after UV.^26^ Furthermore, UV light induces an early type I IFN–dependent migration of inflammatory DCs to SDLNs.^47^ These inflammatory DCs are superior to other DCs for activation of CD4^+^ and CD8^+^ T cells.^48^ We did not observe acceleration of skin lesions in our mice but rather showed that UV light treatment can result in systemic immune activation in an IFNκ‐primed skin environment. We observed enhanced systemic T cell activation even after gross cutaneous lesions had healed. Thus, our data support a role for cutaneous type I IFNs in maintenance of immune activation that could lead to SLE flares, which can be seen after UV treatment.^49^, ^50^

In summary, we describe a new CLE mouse model that recapitulates human CLE lesions and generates systemic immune activation, especially CD8^+^ T cell activation, which can be induced through additional environmental triggers such as UV light. Further research will elucidate the factors that are required for other organ manifestations such as lupus nephritis in mice and humans.

## AUTHOR CONTRIBUTIONS

All authors contributed to at least one of the following manuscript preparation roles: conceptualization AND/OR methodology, software, investigation, formal analysis, data curation, visualization, and validation AND drafting or reviewing/editing the final draft. As corresponding author, Dr Kahlenberg confirms that all authors have provided the final approval of the version to be published and takes responsibility for the affirmations regarding article submission (eg, not under consideration by another journal), the integrity of the data presented, and the statements regarding compliance with institutional review board/Declaration of Helsinki requirements.

## ROLE OF THE STUDY SPONSOR

Eli Lilly, Johnson & Johnson, Bristol Myers Squibb, Sanofi, Prometheus, Almirall, Kyowa‐Kirin, Novartis, AnaptysBio, Boehringer Ingelheim, Regeneron, GSK, AbbVie, and Galderma (support to Dr Gudjonsson), and Q32 Bio, Bristol Myers Squibb, Ventus Therapeutics, ROME Therapeutics, and Johnson & Johnson (support to Dr Kahlenberg) had no role in the study design or in the collection, analysis, or interpretation of the data, the writing of the manuscript, or the decision to submit the manuscript for publication. Publication of this article was not contingent upon approval by Eli Lilly, Johnson & Johnson, Bristol Myers Squibb, Sanofi, Prometheus, Almirall, Kyowa‐Kirin, Novartis, AnaptysBio, Boehringer Ingelheim, Regeneron, GSK, AbbVie, Galderma, Q32 Bio, Ventus Therapeutics, or ROME Therapeutics.

## Supporting information


**Disclosure Form**:

**Data S1** Supporting Information
