# Supplementary material for: Epidermal Interferon‐κ Drives Cutaneous Lupus‐Like Lesions, Photosensitivity, and Systemic Autoimmunity In Vivo
Source: Arthritis Rheumatol. 2025 Nov 27;78(1):184–97. doi: 10.1002/art.43350 (PMC12854000; doi:10.1002/art.43350)
Supplement: Supplementary file 2 — Data S1 Supporting Information [file ART-78-184-s002.docx]

**Supplementary Material:**

**Figure S1. Supplemental data supporting Fig 1**

**Figure S2. Supplemental data supporting Fig 2-3**

**Figure S3: Supplemental data supporting Fig 4**

**Figure S4: Supplemental data supporting Fig 6**

**Supplemental Table 1**

**Supplemental Methods**

**Figure S1.** **Supplemental data supporting Fig 1**


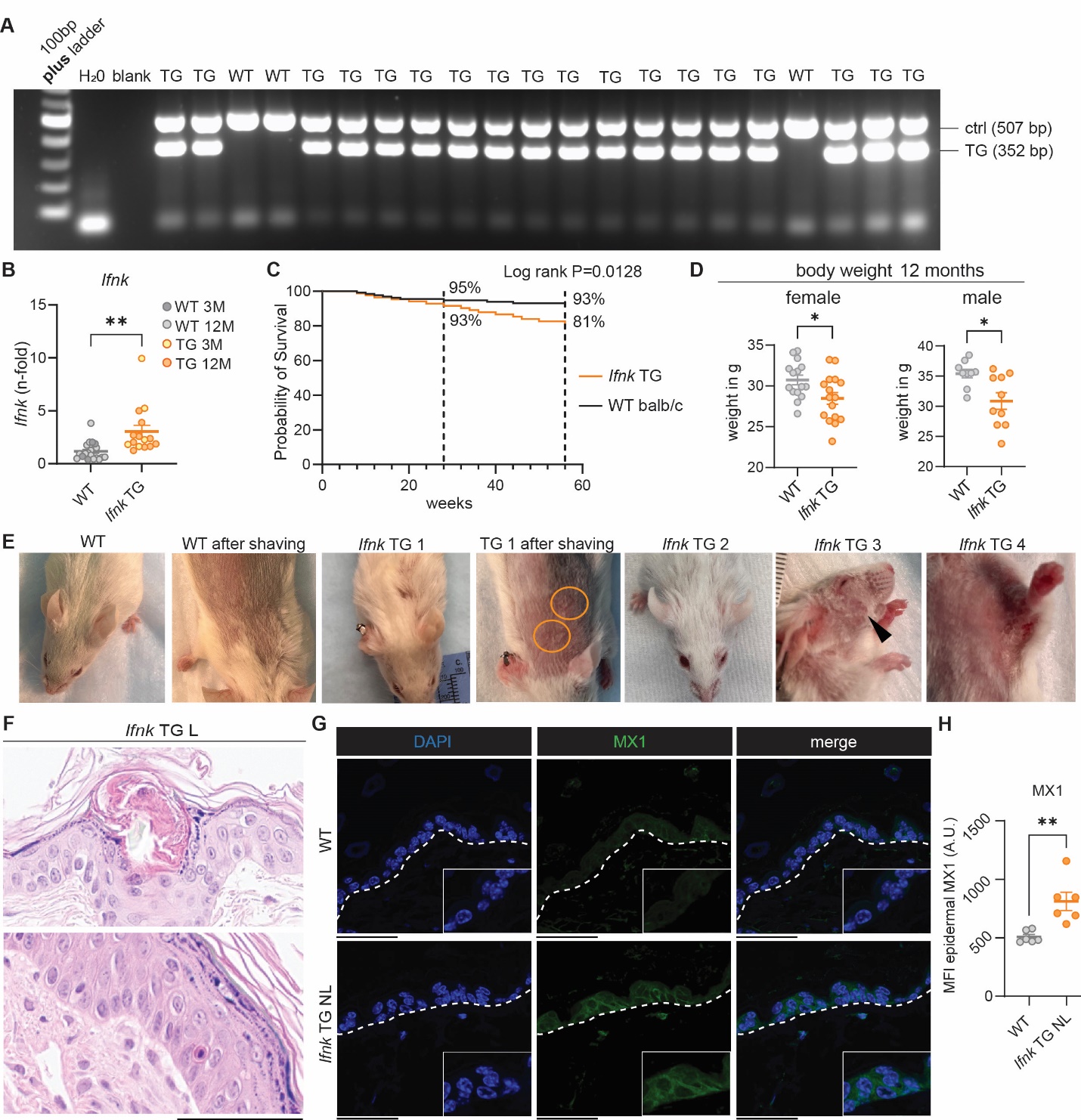


**Figure S1. Supplemental data supporting Fig 1**

**A.** Representative SYBR-stained agarose gel run with PCR-based genotyping results from tail clips or toes from WT and *Ifnk* TG mice. Band at 507 base pairs (bp) indicates the internal control (present in WT and *Ifnk* TG mice) and the band at 352 bp corresponds to the transgene.
**B.** Expression of *Ifnk* in non-lesional skin of WT (n=21) and *Ifnk* TG (n=15) mice. **C.** Survival of WT (n=136) and *Ifnk* TG (n=88) mice over a time course of 56 weeks, Log-rank (Mantel-Cox) test. Dotted lines with percentages of survival are drawn at 28 and 56 weeks. **D.** Comparison of body weights in female (left) and male (right) WT and *Ifnk* TG mice at 12 months of age, n=9-16 per group. **E.** Representative images of WT before and after shaving and different *Ifnk* TG mice before and after shaving. Note scaly plaques on the back of *Ifnk* TG1 after shaving. *Ifnk* TG 2 shows periorbital swelling and erythema. *Ifnk* TG 3 shows a facial erythematous, hairless plaque. *Ifnk* TG 4 exhibits erythematous, hairless plaque in the axillary region. **F.** Representative H&E-stained skin lesions from *Ifnk* TG mice harboring follicular plugging (top) and necrotic epidermal KC (bottom), scale bar=60μm. **G.** Representative immunofluorescence images of WT and *Ifnk* TG nonlesional (NL) skin stained for DAPI (blue) and MX1 (green), scale bar=50μm. Dotted line indicates dermal-epidermal junction and white boxes represent zoomed-in images. **H.** Quantification of epidermal mean fluorescence intensity of MX1 in WT and *Ifnk* TG NL skin (n=6 each). Student’s t-test (B, D), Mann Whitney U test (H). *P<0.05, **P<0.01.

**Figure S2. Supplemental data supporting Figs 2-3.**

**
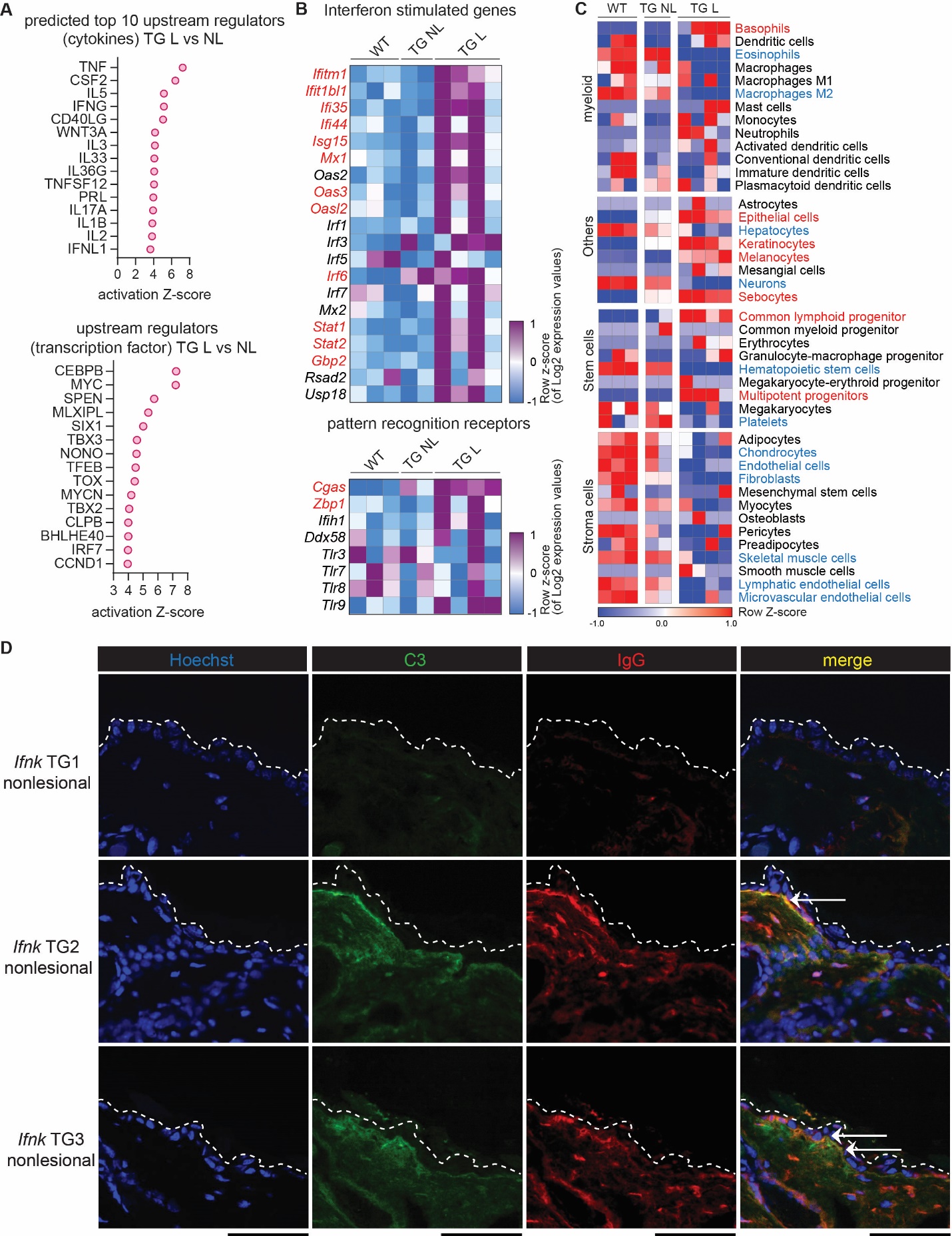
**

**Figure S2. Supplemental data supporting Figs 2-3.**

**A.** Dot plot of the top 20 predicted upstream regulators split by cytokines (left) and transcription factors (right) enriched in DEGs in *Ifnk* TG L skin versus *Ifnk* TG NL skin. **B.** Heatmaps of selected genes with row z-scores of log2 expression values of IFN-stimulated genes and pattern recognition receptors in WT, *Ifnk* TG NL and *Ifnk* TG L skin. Red colored font indicates significant upregulation in *Ifnk* TG lesional skin vs WT, whereas blue colored font indicates significant downregulation in *Ifnk* TG lesional skin vs WT. **C.** Heatmap of XCell analysis representing XCell-derived row Z-scores for myeloid cells, other cell types, stem cells and stromal cells in WT, *Ifnk* TG NL and *Ifnk* TG L skin. **D.** Representative immunofluorescence images of nonlesional skin taken from back skin in *Ifnk* TG mice stained for C3 (green), IgG (red) and nuclei (Hoechst). Dotted line indicates the upper border of the epidermal cell layer. Arrows indicate positive staining at dermal-epidermal junction in TG2 and TG3. Scale bar = 50μm.

**Figure S3: Supplemental data supporting Figure 4.**


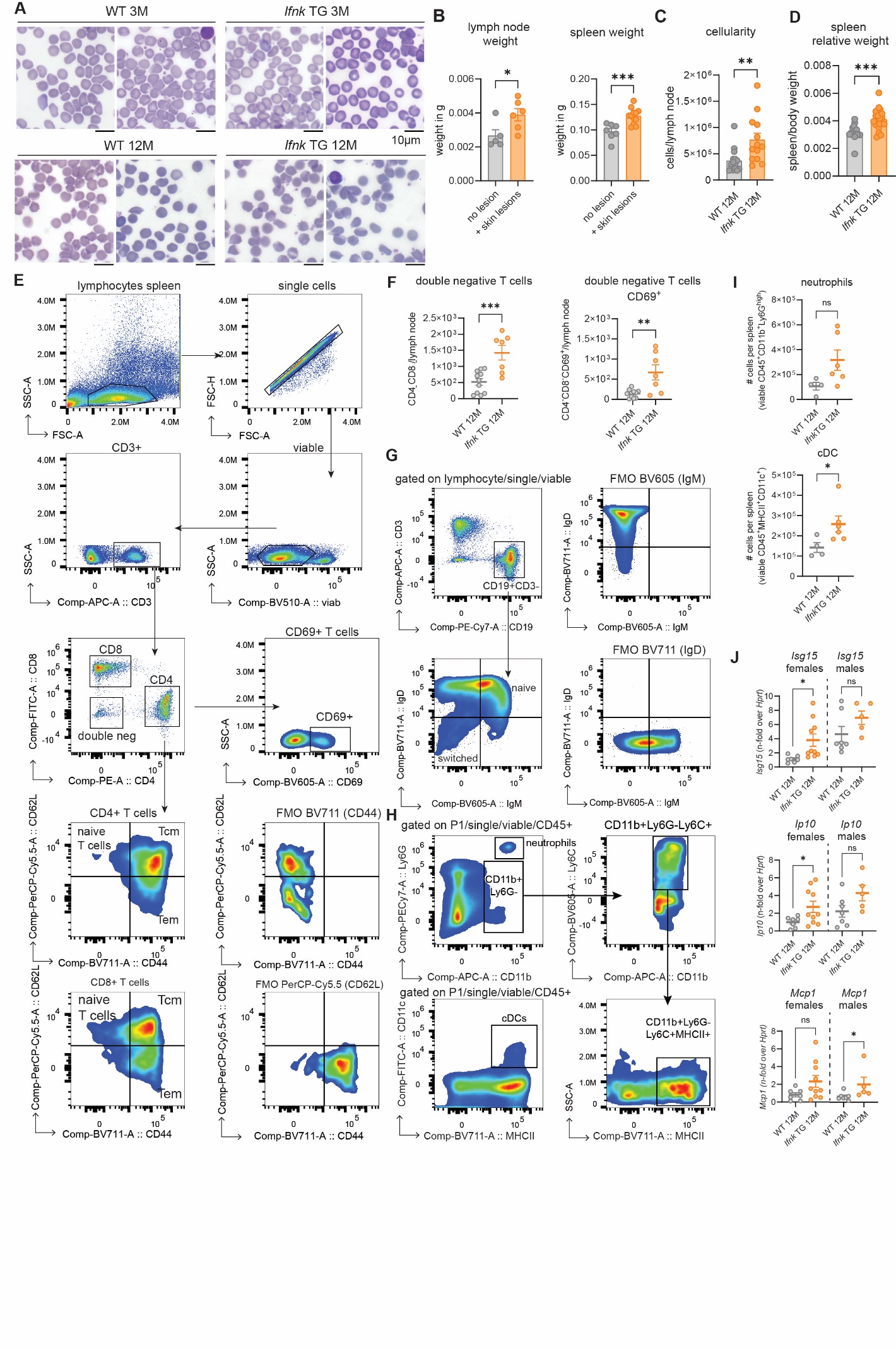


**Figure S3: Supplemental data supporting Figure 4.**

**A.** Representative images of blood smears obtained from peripheral blood from young and aged WT and *Ifnk* TG mice reviewed by an animal pathologist. No evidence of corpuscular hematolytic anemia, schistocytes, spherocytes or other abnormalities in red blood cells. Samples show slight post-collection artifactual changes like echinocyte formation, not pathogenic. No morphological differences in white blood cells. Adequate platelet count in all samples (n=2 for each strain and age), scale bar 10μm. **B.** Comparison of lymph node and spleen weights in *Ifnk* TG mice without lesions (no lesion) or with skin lesions (+ skin lesion). **C.** Bar plot comparing total cell counts in SDLN of WT and *Ifnk* TG mice. **D.** Bar plot comparing spleen weights in WT and *Ifnk* TG mice.
**E.** Gating strategy for T cell panel in splenocytes. T cells were gated using lymphocyte/single/viable/CD3^+^ cells and assessed for expression of CD4 and CD8. Further gating included CD69. Gating of activation markers CD44 and CD62L was based on fluorescence minus one (FMO) controls for both markers. **F.** Scatter plots with comparison of double negative T cells and activated double negative T cells (CD69^+^) in lymph nodes of WT and *Ifnk* TG mice. **G.** Gating strategy for switched B cells based on expression of IgM and IgD using FMO controls for both markers. **H.** Gating strategy for myeloid cells gated on single/viable/CD45^+^. CD45^+^CD11b^+^Ly6G^-^ cells were further gated for expression of Ly6C and MHCII. **I.** Scatter plots compare total splenic neutrophils (CD11b^+^Ly6G^+^) and cDCs (CD11c^+^MHCII^+^) in WT and *Ifnk* TG mice. **J.** Expression of *Isg15*, *Ip10* and *Mcp1* in male and female WT and *Ifnk* TG mice highlighting sex differences in ISG and chemokine expression. Student’s t-test or Mann-Whitney U test. *P<0.05, **P<0.01, ***P<0,001.

**Figure S4: Supplemental data supporting Figure 6:**


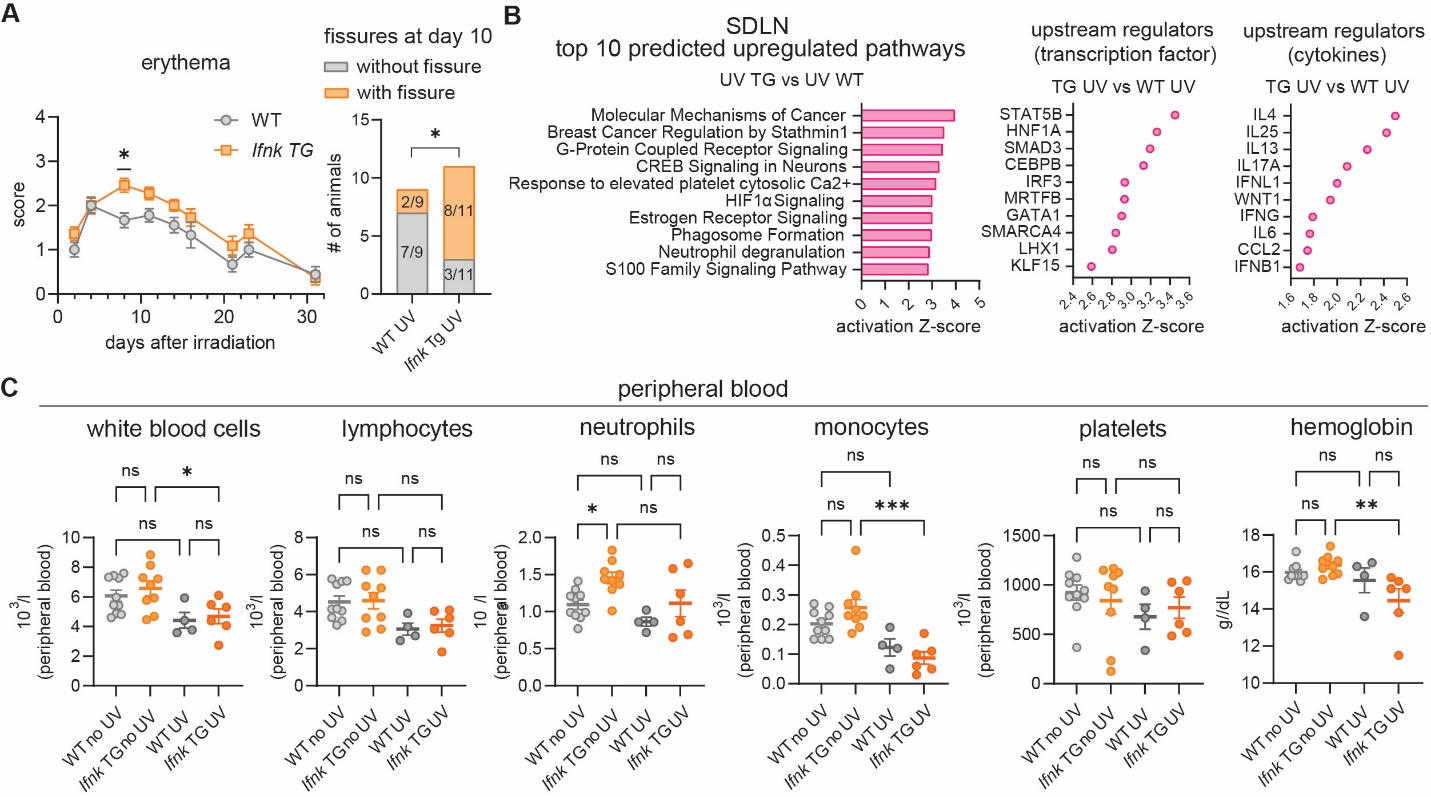


**Figure S4: Supplemental data supporting Figure 6.**

**A.** Erythema over time and comparison of fissures at day 10 after irradiation in WT and *Ifnk* TG mice treated with UV light. Clinical severity score in Fig 5C was based on erythema and presence of fissures. When a fissure was present, one extra point was given for comparison in Fig 5C. Two-way ANOVA followed by Sidak’s multiple comparisons test (left) and Chi^2^ Test. **B.** Left: Bar plots representing the activation Z-scores of the top 10 immune-related pathways enriched among DEGs in SDLN of UVB-treated *Ifnk* TG mice (n=3) vs WT mice (n=3). Middle and right: Dot plots showing the top 10 upstream regulators (middle: transcription factors, right: cytokines) enriched among DEGs in SDLN of UV-treated *Ifnk* TG mice vs WT mice. **C.** Scatter plots compare peripheral blood counts in WT and *Ifnk* TG mice at baseline and after UV exposure (2 weeks after observation period). One-way ANOVA followed by Sidak’s multiple comparisons test. n=4-10 per group. *P<0.05, **P<0.01, ***P<0,001.

**Supplemental Table 1: Antibodies used for flow cytometry and tissue staining**

| **Antibody** | **Source** | **Identifier** |
| --- | --- | --- |
| TruStain FcX (anti-mouse CD16/32) | Biolegend | #101320 |
| Brilliant Violet 510 Ghost Dye | Cytek Biosciences | #13-0870-T100 |
| Brilliant Violet 605 anti-mouse Ly6C Clone HK1.4 | Biolegend | #128036 |
| Brilliant Violet 711 anti-mouse I-A/I-E | Biolegend | #107643 |
| PerCP anti-mouse CD45 | Biolegend | #103130 |
| PE/Cyanine7 anti-mouse Ly-6G | Biolegend | #127618 |
| FITC anti-mouse CD11c | Biolegend | #117306 |
| APC anti-mouse/human CD11b (M1/70) | Biolegend | #101212 |
| APC anti-mouse CD3 (17A2) | Biolegend | #100236 |
| FITC anti-mouse CD8a | Biolegend | #100706 |
| PerCP/Cyanine5.5 anti-mouse CD62L | Biolegend | #104432 |
| PE anti-mouse CD4 Antibody anti-CD4 - RM4-5 | Biolegend | #100512 |
| BV605 anti-mouse CD69 Clone H1.2F3 | Biolegend | #104530 |
| Brilliant Violet 711 anti-mouse CD44 | Biolegend | #103057 |
| PE anti mouse CD45R/B220 | Biolegend | #103208 |
| Brilliant Violet 605 anti-mouse IgM | Biolegend | #406523 |
| Brilliant Violet 711 anti-mouse IgD | Biolegend | #405731 |
| PE/Cyanine7 anti-mouse CD19 | Biolegend | #115520 |
| Rabbit anti-CD4 [EPR19514] | Abcam | ab183685 |
| Rabbit anti-CD8 [EPR21769] | Abcam | ab217344 |
| Rabbit anti-CD11b [EPR1344] | Abcam | ab133357 |
| Rabbit anti-MX1 (D3W71) | Cell signaling | #37849 |
| Goat anti-mouse IgG Texas Red | Invitrogen | T-862 |
| Goat anti-Mouse C3 - FITC Conjugated | ICL | GC3-90F-Z |

**Supplemental Table 2. Mouse primers**

| **Gene** | **Forward sequence 5’ 3’** | **Reverse Sequence 5’ 3’** |
| --- | --- | --- |
| *Isg15* | CAGAAGCAGACTCCTTAATTC | AGACCTCATATATGTTGCTGTG |
| *Ip10* | ATCATCCCTGCGAGCCTAT | ATTCTTGCTTCGGCAGTTAC |
| *Mcp1* | AGGTCCCTGTCATGCTTCTG | TCTGGACCCATTCCTTCTTG |
| *Hprt* | GAGAGCGTTGGGCTTACCTC | CTAATCACGACGCTGGGACT |

**Supplemental Methods:**

**Harvest procedure:**

Mice at their specified timepoint (3 months, 4 weeks post UV-B and 12months) were anesthetized with an intraperitoneal injection of sodium pentobarbital (either 100 mg/kg or 125 mg/kg). In complete absence of a pain response, the abdominal cavity was opened and the spleen removed, measured, weighed and cut into sections for flow cytometry, RNA extraction, and histology. Before kidney removal, the kidneys were flushed with heparin (60mg dissolved in 300mL PBS), via a cardiac puncture of the beating heart, until they turned pale and were void of blood. One kidney was used for flow cytometry and the other kidney was cut into sections for RNA extraction, histology (both formalin fixed and OCT, optimum cutting temperature freezing medium). Inguinal lymph nodes were removed, measured, weighed, and processed for flow cytometry or RNA extraction. Lesional skin (wherever present) was removed for histology, and if enough present, RNA extraction and OCT. Non-lesional skin was harvested from the back and comparable areas to where lesional tissue was located for histology (FFPE), RNA extraction and OCT.

**RNA extraction**

For isolation of RNA from skin tissue, frozen samples were crushed with mortar and pestle prior to homogenization using a handhold tissue homogenizer (Grainger). For lymph node RNA isolation, inguinal skin-draining lymph nodes were put through a 70μm cell strainer. Homogenates were then lysed in RLT lysis buffer and RNA was purified using RNeasy Plus Mini Kit (Qiagen). For isolation of splenic RNA, we used ¼ of the whole spleen and the RNeasy Mini Kit (Qiagen, Cat. No. 74106). Each sample was lysed in RLT lysis buffer and after adding buffer RW1 and centrifugation, samples were subjected to DNA digestion using RNAse-free DNAse Set (Qiagen, 79524) with 10uL DNase I to 70uL Buffer RDD for each sample for 15min at RT. Next, RNA isolation was continued according to the RNeasy Mini Kit using buffer RW1 and RPE. Total RNA quantity and purity were determined based on A260nm/A280 nm using a BIOTEK EPOCH plate reader.

**cDNA synthesis and quantitative PCR (qPCR)**

After isolation of RNA, a total of 200 to 1000ng of cDNA was synthesized using the iScript cDNA synthesis kit (BioRad, #1708891). 10ng cDNA was used for quantitative PCR in technical triplicates in a 384 well plate using SYBR Green Supermix (Applied Biosystems). See Supplemental Table 2 for primers used. PCR was run in Applied Biosystems QuantStudio™ 12K Flex Real-Time PCR at the Advanced Genomics Core at the University of Michigan. Primer sequences are summarized in Supplemental Table 2. Gene expression level was determined by relating to the housekeeper gene *Hprt* using the ∆∆ct method setting the corresponding sex-specific WT condition to one.

**Statistics**

Graphing and statistical analysis of all data was performed using GraphPad Prism v.10 or R. Where appropriate, one-way or two-way ANOVA was used to compare multiple groups, and a two-tailed Student's t-test for normally distributed data was used for comparison between two groups. For comparison of two groups with non-normally distributed values, a Mann-Whitney U test was used. P-values less than 0.05 were considered significant. We performed RNA sequencing analyses of skin and lymph nodes using the DESeq2 package in R, where an FDR-adjusted p-value less than 0.05 was considered statistically significant. IPA Canonical Pathways and Upstream Regulator analyses were then performed and the significant ones with a Z-score of ≥ 2 or ≤ -2 were filtered out (see detailed description in method section ‘RNA Sequencing, Analysis and XCell enrichment tool’).

**Immunohistochemistry**

For detection of CD4 (1:1000), CD8 (1:2000), CD11b (1:1000) in the skin, FFPE sections were heated at 60°C for 1 hour, deparaffinized, rehydrated, and antigen retrieval was performed using a pressure cooker and antigen retrieval buffer of pH=9 for CD4, CD8, CD11b and CD11c). Slides were washed, treated with 3% hydrogen peroxide in PBS for 5 minutes, blocked (10% goat serum), and incubated with primary antibodies overnight at 4°C. All slides were then incubated with for 30 minutes with goat anti-rabbit IgG (H+L) biotinylated secondary antibody (1:200), followed by incubation with Vectastain Elite ABC-HRP reagent R.T.U. (Vector Laboratories, SK-4105), followed by detection using DAB substrate (BD Pharmingen, #550880) under a light microscope. Slides were then counterstained with hematoxylin, neutralized in bluing reagent, dehydrated, and mounted. Images were obtained through scanning of stained slides through the UMich Pathology slide scanning core.

**Immunofluorescence**

For staining of IgG and C3, we used frozen sections from skin and kidneys. Slides were dried for 1h at 37C, fixed with 4% PFA, washed and blocked with 10% normal goat serum for 1h before incubation for 30min with primarily conjugated antibodies against C3 (goat anti-C3 FITC conjugated, Immunology Consultants, GC390FZ, 1:250) and IgG (goat anti-Mouse IgG (H+L) Cross-Adsorbed Secondary Antibody, Texas Red-X, Invitrogen, T-862, 1:250) in blocking solution. After washing, slides were incubated with Hoechst33342 for 5min, washed, mounted and images were taken the next day using a NikonYokogawa Spinning disk microscope. For each sample, three images were taken based on Hoechst staining to identify skin/kidney structures and to reduce operator bias. Immunofluorescence of MX1 was performed on FFPE slides of WT and *Ifnk* TG nonlesional skin. Samples were antigen-retrieved and blocked as described above (see Immunohistochemistry) and incubated with rabbit anti-MX1 (Invitrogen 13750-1-AP, 1:200) overnight. Secondary antibody (goat anti-rabbit, Alexa Fluor Plus 488 (Invitrogen 11008, 1:500) and DAPI (1:500) for 30min. Slides were then washed and mounted using ProLong Glass Antifade Mountant (Invitrogen, P36980). ImageJ was used for quantification of epidermal mean fluorescence intensity of MX1 in three independent regions of interest based on DAPI-staining.

**Complete blood count and serum:**

Submandibular blood for CBC was collected in EDTA tubes and analyzed at our ULAM Pathology Core with an ElementHT5 Analyzer. For serum, tube with dislodged clot was centrifuged at 4°C for 5 min at 10000xg. Serum was stored at -80°C until processed.

**Albumin/Creatinine ratio, anti-dsDNA antibodies and total serum IgG**

Albumin and creatinine were quantified using ELISA kits (Ethos Biosciences #1011 and Ethos Biosciences #1012) according to manufacturer’s instructions. Urine samples were collected from live mice and stored at -20°C. Samples were thawed and diluted 1:20 in NHEBSA (albumin) or water (creatinine). Anti-dsDNA and IgG levels were analyzed with the use of ELISA kits (Alpha Diagnostic, San Antonio, TX, # 6320 – Ms IgG and #5120 – Ms dsDNA), according to the manufacturer’s instructions. For anti-dsDNA antibodies, we used 1:500 dilutions and for IgG 1:50,000. All values were obtained using microplate reader according to the manufacturer.
